# Stress and behavioral correlates in the head-fixed method: stress measurements, habituation dynamics, locomotion, and motor-skill learning in mice

**DOI:** 10.1038/s41598-020-69132-6

**Published:** 2020-07-22

**Authors:** Konrad Juczewski, Jonathan A. Koussa, Andrew J. Kesner, Jeong O. Lee, David M. Lovinger

**Affiliations:** Section on Synaptic Pharmacology and In Vivo Neural Function, Laboratory for Integrative Neuroscience, National Institute on Alcohol Abuse and Alcoholism, US National Institutes of Health, Rockville, MD 20852 USA

**Keywords:** Behavioural methods, Stress and resilience

## Abstract

Manual restriction of head movement, or head-fixation, of awake rodents allows for sophisticated investigation of neural circuits in vivo, that would otherwise be impossible in completely freely moving animals. While it is known that head-fixation induces stress, the scale of this stress and habituation dynamics remain unclear. We used the Mobile HomeCage system (Neurotar Ltd, Finland) where animals have their heads fixed to an aluminum frame but are otherwise freely moving in an ultralight carbon container floating above an air-dispensing base. For 25 consecutive days, mice were head-fixed while standing on the air-lifted platform for 2 h per day and blood samples were taken periodically to measure variation in the stress-related hormone, corticosterone. We showed that the initial increase in corticosterone concentration is followed by a return to control level throughout the days of head-fixed training. We also found a locomotor correlate of this drop. We conducted a battery of stress-sensitive behavioral paradigms in freely-moving mice that revealed minor differences following chronic head-fixation. Finally, we analyzed motor-skill learning in the head-fixed setup with a floating container. We believe that our results may contribute to better interpretation of past literature and future in vivo experiments using head-fixed animals.

## Introduction

Stress can have profound effects on both an animal’s behavior^1,2^ as well as physiology^3,4^. Therefore, understanding how stress might factor into awake-behaving animal experiments is important for interpreting results and making valid conclusions. This is particularly relevant when adopting new techniques, as the levels of stress induced by these techniques has often not yet been systematically examined and, rather, has been suggested only anecdotally when compared to more established behavioral paradigms. One such technique is the securing of the head of an unanesthetized animal, or head-fixing, to avoid effects of head movement during behavioral and neurophysiological experiments. Although head-fixation has been used for several decades in neurophysiological studies in monkeys^5,6^, it is only more recently that this technique has been gaining popularity in neurobiological studies using rodents, most typically mice. This renaissance was sparked by new experimental environments and modern neurobiological techniques such as optogenetics and high-resolution brain imaging^7,8^. By combining these approaches with head-fixation, investigators have been able to elucidate neural mechanism of behavioral processes at unprecedented biological resolution.

What is common for all the standard head-fixation techniques is a metal plate surgically placed on the mouse’s head such that the plate can then be attached with screws to a set of clips that restrict head-movement. However, a variety of head-fixation techniques can be distinguished based on the extent of body and head immobilization. One type of technique is to fully restrain the torso of the animal after head-fixation^9,10^. It is a simple, compact and affordable system. However, it creates a restricted environment for mice that is likely anxiogenic and reduces the behavioral repertoire available for analysis and interpretation of results. A second type of head-fixation technique involves free running on a treadmill or a spinning disc^7, 11^. It allows for limb movement and some aspects of locomotor behavior but forces the animal to an unnatural body alignment and limits its possible movements to linear acceleration and deceleration. Building further is the floating-ball approach, where the mouse can move on top of a spherical ball lifted by air^12,13^. This arrangement allows a greater range of movement, but the body posture is still uncomfortable and the whole system is large and bulky. Finally, the newest approach involves a system called the mobile homecage (MHC)^14,15^, which is comprised of an air-lifted platform placed under the head-fixation frame. In contrast to other systems, it constitutes a less restricted environment giving the mouse the opportunity for body movements in multiple directions and it allows the mouse to keep more natural body posture. The MHC system is also quite compact compared to floating-ball techniques and can be easily incorporated into existing experimental set-ups and apparatuses. While the MHC system provides a more naturalistic environment that makes results more relatable to freely moving experiments, past studies using the MHC system have not provided in-depth analysis of indices of stress produced by this head-fixation technique, so the generalization of conclusions made using this technique is unclear. The present study aimed to fill this knowledge gap by systematically measuring levels of corticosterone, a stress hormone that is a common index of an animals perceived stress level^4^, and also examining stress-related behavioral phenotypes during a 25-day head-fixed protocol.

## Results

### Preparation for the head-fixed experiments

**Figure 1:**
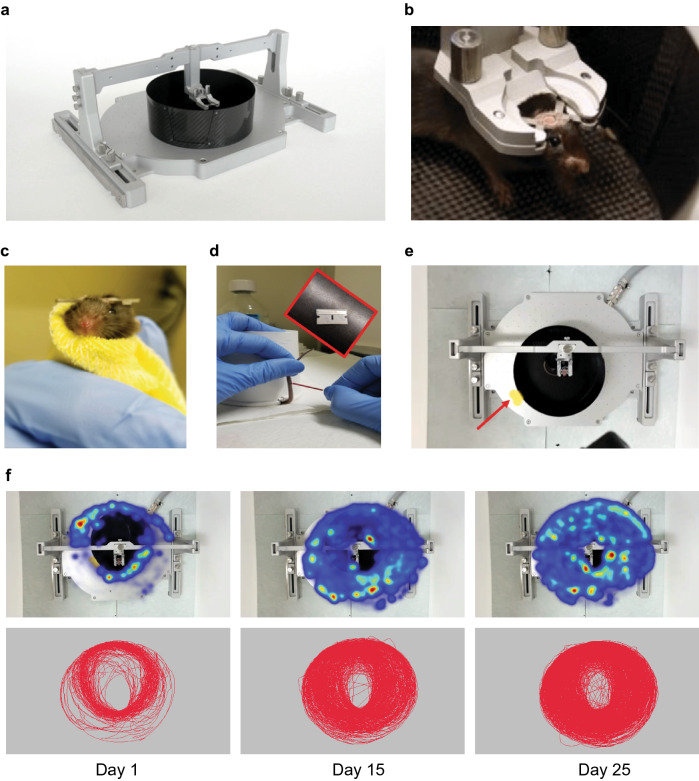
Overview of the methods. (**a**) Head-fixed apparatus: the air-lifted carbon fiber container (in black) placed under the head-fixation frame on the air-dispensing base. (**b**) Mouse with surgically attached head-plate fixed to the head-plate holder in the head-fixation frame. (**c**) Mouse wrapped in flannel for transfer between the home cage and the head-fixed apparatus. (**d**) Mouse partially restrained with a paper cup covering its body with the tail kept outside of the cup ready for blood sampling collection with a glass capillary after a tail-snip with a razor blade. (**e**) Video frame from the camera mounted on the top of the head-fixed apparatus to collect information for the locomotion analysis. Red arrow points at the yellow sticker attached to the floating container that was tracked with the EthoVision software for the movement analysis. (**f**) Example of heatmaps (top panel) and track maps (bottom panel) extracted with the software for locomotion analysis from the recorded videos at day 1, day 15 and day 25.

We first developed a standardized method for habituating mice to head-fixation and the MHC apparatus (Fig. 1a, b), based on prior work from Kislin et al.^14^. In our experiments, we aimed to measure the initial level of stress in the head-fixed situation and if stress indices are reduced after habituation to head-fixation. All the animals (the head-fixed and the controls) used in all the protocols underwent the head-plate attachment surgery followed by 2 days of post-operative care. A week after the surgery every animal was habituated in two 15-min sessions (one session a day), first to the experimenter, then to the flannel that was being used to transfer animals between the home cage and the head-fixed apparatus (Fig. 1c). Finally, throughout the days of the different head-fixed protocols, mice were placed in the head-fixed apparatus for 120 min every day with the floating container underneath. We took blood samples from the tail vein at the end of the head-fixed session every 5 days (Fig. 1d) and measured variations in blood concentrations of the stress-related hormone, corticosterone (for review, see^4^). The control animals were wrapped in the flannel for only about a minute and left in the cage in the same room as their head-fixed littermates for the time of the head-fixed session.

Preparatory experiments were performed on mice generated by crossing the Emx1::Cre animals with the Ai32 channel rhodopsin line, animals that were readily available in our laboratory at the time. In the 10-day preparatory protocol, the mice were head-fixed 10 times for 120 min/day and blood samples were collected at the end of the session on days 1, 5 and 10 (Supplementary fig. S1a). We found a significant group effect: two-way RM ANOVA, interaction, F(2, 8) = 0.8942, *p* = 0.4462; time F(1.179, 4.678) = 4.678, *p* = 0.1001; group F(1, 4) = 149.7, *p* = 0.0003. Initially, increased corticosterone level in the head-fixed group that was higher than the control group (151.50 ± 21.40 vs. 43.03 ± 2.95 on day 1) was reduced over the 5-day habituation period (to 119.0 ± 10.04), and the full 10-day protocol reduced it even further (to 80.44 ± 23.54) (Supplementary fig. S1b). We also ran a 5-day preparatory study in which blood sampling was performed 3 times (0, 60 and 120 min) per every head-fixed session (Supplementary fig. S1c). In the head-fixed animals, we observed a clear increase in the blood corticosterone concentration 60 min after the initial blood sampling (410.4 ± 17.39% of the time 0 min) through the end of the session at 120 min (379.0 ± 35.77%) (Supplementary fig. S1d–g). In contrast, we did not see any large changes in the non-head-fixed control animals (100.9 ± 38.29% and 95.33 ± 8.721%, respectively) in which the corticosterone level continued to be low. Based on these promising results from our preparatory experiments, we ran an extended 25-day habituation protocol to observe further habituation dynamics. We decided to run 120-min head-fixed sessions using the mouse strain most commonly used in neurobiological experiments, C57Bl6/J^16–18^. We also chose to collect blood samples every 5 days to avoid problems related to potential tail injuries due to frequent tail snips.

### Decrease of the head-fixation-related corticosterone level and weight loss in the extended 25-day habituation protocol

**Figure 2:**
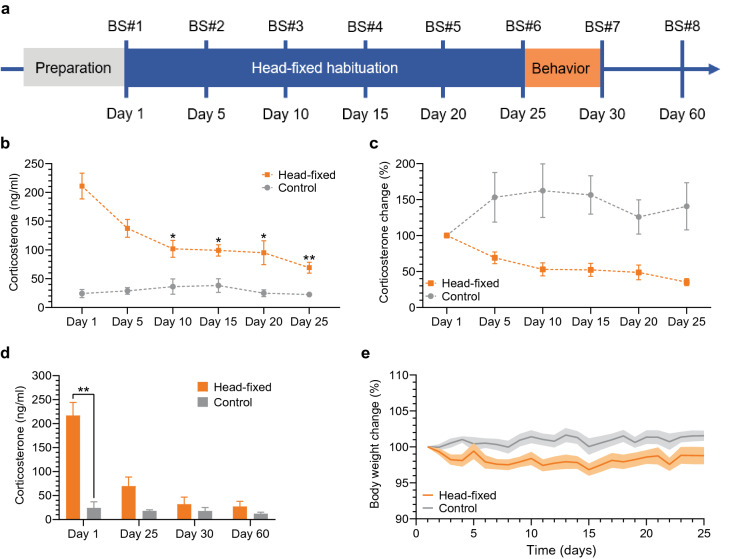
Corticosterone and body weight dynamics in the 25-day head-fixed protocol. (**a**) Timeline of the experiments; blood sampling (BS) every 5 days (BS#1, BS#2, etc.) at the end of the 120-min head-fixed session except for BS#7 and BS#8 (no head-fixing, only BS). (**b**) Increased blood corticosterone concentration in the head-fixed group in comparison to the low and stable corticosterone level in the control group: two-way RM ANOVA, interaction, F(5, 70) = 11.90, *p* < 0.0001; time, F(2.326, 32.57) = 10.76, *p* = 0.0001; group, F(1, 14) = 54.11, *p* < 0.0001. Consistent drop in the corticosterone level in the head-fixed group: Bonferroni’s post hoc tests, within-group comparisons with day 1 (D1); *p < 0.05; **p < 0.005. (**c**) Percentage change in the blood corticosterone concentration normalized to D1; n = 8 in each group. (**d**) Significant difference in the blood corticosterone concentration between the head-fixed and the control groups on D1 became non-significant at D25 of the head-fixed habituation protocol and stayed at the lower level after the last day of head-fixing: two-way RM ANOVA followed by Bonferroni’s post hoc tests (between groups), interaction, F(3, 18) = 16.53, *p* < 0.0001; time effect, F(1, 6) = 53.64, *p* = 0.0003; group effect, F(1.660, 9.963) = 20.07, *p* = 0.0005; the head-fixed vs. the control group on D1 (**p = 0.0095) and on other days (D25, D30, D60, *p* > 0.2837); n = 4 in each group. (**e**) Changes in body weight during the 25-day protocol expressed as the percentage of the weight at D1. Weight drop in the head-fixed group leading to a statistically significant difference in comparison to the control group starting on day 3 and lasting until the end of the protocol: two-way RM ANOVA followed by Bonferroni’s post hoc tests (between groups): interaction, F(24, 168) = 1.493, *p* = 0.0756; time, F(1, 7) = 5.729, *p* = 0.0479; group, F(24, 168) = 2.043, *p* = 0.0048; significant difference in all the post hoc comparisons between the groups except for D1, D2 and D5 (*p* > 0.9999); *p < 0.05; **p < 0.01; n = 8 in each group.

After the preparation phase that consisted of the head-plate surgery, 7 days of recovery and 2 days of handling, we ran the extended 25-day habituation protocol. It was followed by 4 days of behavioral testing in classic stress-sensitive paradigms (a timeline is presented in Fig. 2a). In the 25-day protocol we found that the initial blood corticosterone concentration after the first head-fixed session was 210.9 ± 22.46 ng/ml, a level that was about 9 times higher than the baseline level measured in the control animals, 24.2 ± 7.05 ng/ml (Fig. 2b). Corticosterone level decreased consistently over the course of the habituation protocol in the head-fixed group and remained stable at the low level in the control group (note that fluctuations observed in the control group are not statistically significant). Accordingly, two-way RM ANOVA analysis revealed an interaction between group and time: F(5, 70) = 11.90, *p* < 0.0001; as well as effects of time F(2.326, 32.57) = 10.76, *p* = 0.0001; and group F(1, 14) = 54.11, *p* < 0.0001. Furthermore, post hoc Bonferroni’s tests for within-group comparisons showed that the first significant decrease in the corticosterone level occurred at day 10 of the habituation protocol (day 1 vs. day 10, *p* = 0.0426). Also, on every subsequent blood sampling day, the blood corticosterone level dropped further, and each measurement was significantly lower than on day 1 (day 1 vs. day 15, day 20 and day 25, *p* < 0.05). In fact, the three largest drops in the corticosterone level in the head-fixed group were between days 1 and 5 (reduction to 69 ± 8.091% of day 1 levels), between days 5 and 10 (down to 52.97 ± 8.997%), and between days 10 and 25 (down to 35.22 ± 4.988%) (Fig. 2c). We stopped head-fixing animals on day 25, however, in four cases we still measured corticosterone concentration on subsequent days. Two-way RM ANOVA analysis revealed again an interaction between group and time: F(3, 18) = 16.53, *p* < 0.0001; an effect of time F(1, 6) = 53.64, *p* = 0.0003; and group F(1.660, 9.963) = 20.07, *p* = 0.0005. Afterwards post hoc Bonferroni’s tests were run, this time for comparisons between the groups. These tests showed a significant difference at day 1 (*p* = 0.0095) and no statistically significant differences at days 25, 30 and 60 (*p* > 0.05). Blood corticosterone concentration in the head-fixed group decreased further after the last day of head-fixing from 69.7 ± 18.91 ng/ml (day 25) to 32.0 ± 14.65 ng/ml (day 30) and stayed at this level through day 60 (27.2 ± 10.82 ng/ml) (Fig. 2d).

Chronic stress in rodents may result in decreased food intake leading to weight loss^3,19,20^. Therefore, we also recorded the weight of the head-fixed and control animals before every head-fixed session. In adult mice (after about 12 weeks of age) weight is stable and increases at a very slow pace^21^. This was observed in our control group: the average weight oscillated around 30.7 ± 0.03 g, but it slightly increased from day 1 (30.43 ± 0.63 g) to day 25 (30.91 ± 0.73 g) of the studies. In contrast, in the head-fixed group, the average weight oscillated around 29.5 ± 0.04 g, but it decreased with time from day 1 (30.0 ± 0.60 g) to day 25 (29.6 ± 0.62 g). Two-way RM ANOVA analysis showed no interaction between group and time: F(24, 168) = 2.043, *p* = 0.0756; an effect of time: F(1, 7) = 5.729, *p* = 0.0479; and group: F(24, 168), *p* = 0.0048. Bonferroni’s post hoc tests run for comparisons between the groups revealed that the weight drop visible in the head-fixed group after the first head-fixed session led to a significant difference between the groups already at day 3 (*p* = 0.0457, 98.2 ± 0.65% vs. 100.5 ± 0.5% of the initial body weight, the head-fixed and the control group, respectively; Fig. 2e). The difference in weight between the groups, despite some minor fluctuations, remained stable till the end of the 25-day protocol (comparison of the head-fixed vs. the control group from day 3 to day 25, *p* < 0.05 for all the comparisons except for day 5, *p* > 0.9999).

Corticosterone secretion is influenced by various external and internal factors; therefore, we conducted control experiments and additional analyses. Firstly, we checked whether the blood sampling procedure in itself constituted a confounding factor in our experiments due to shifting the baseline corticosterone level. We ran 3 blood sampling control experiments in which animals underwent the same initial procedures as the animals in the 25-day protocol, but were withdrawn from the study after a single blood sampling at days 5, 15 or 25. In the head-fixed group we did not observe any difference in the corticosterone level between those animals and the corresponding animals that had been blood-sampled several times during the 25-day protocol (Supplementary fig. S1h). However, in the control group, a similar comparison showed a higher level of corticosterone at days 5 and 15 in the animals that had been blood-sampled several times (Supplementary fig. S1i). Moreover, knowing that in rodents the corticosterone level follows a circadian rhythm^22,23^, we counter-balanced our experimental groups in the 25-day protocol. We chose 4 time points for the head-fixed sessions and we looked at the blood sampling results in that context. We saw the expected increase in the blood corticosterone concentration throughout the day, with major differences between the morning- and the late afternoon-sampled animals (Supplementary fig. S1j). In contrast, in the head-fixed group, this pattern was interrupted. To sum up, control experiments/analysis showed that less frequent blood sampling (every 5 days) and counter-balancing the groups in terms of the time of day of the experiment were important for an optimal experimental design.

### Repeated head-fixation does not alter several stress-sensitive behavioral phenotypes, but results in altered fluid intake

**Figure 3:**
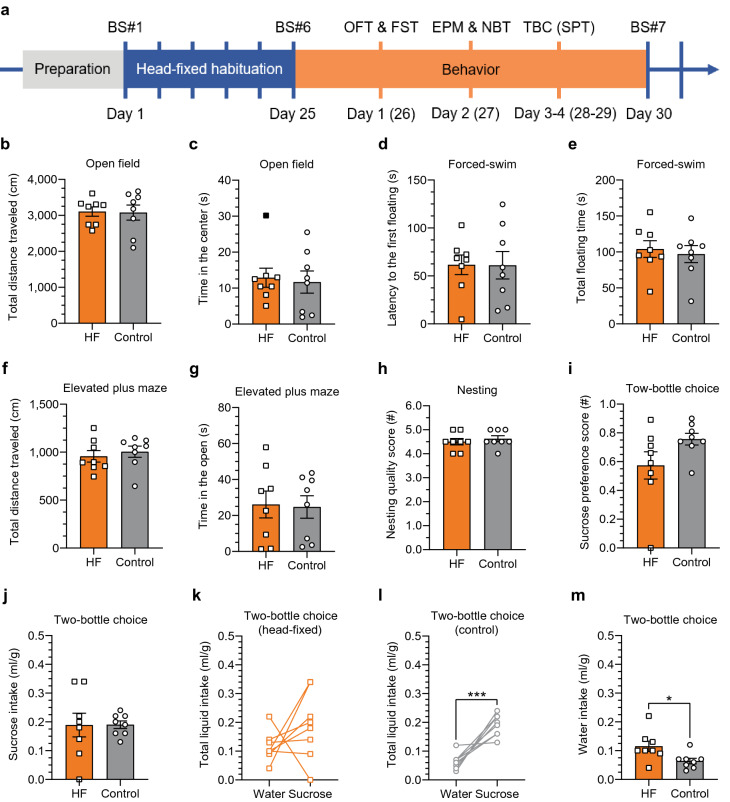
Long-term effects of the 25-day head-fixation tested in stress-sensitive behavioral paradigms. (**a**) Detailed timeline of the behavioral experiments that followed 25-day head-fixed habituation with blood sampling (BS) every 5 days (BS#1 etc.). Behavioral paradigms included: the open field test (OFT); the forced-swim test (FST); the elevated plus maze (EPM), nesting behavior (NB), and the two-bottle choice/sucrose preference test (TBC/SPT). (**b**, **c**) Both groups traveled similar distances during the trial and spent similar time in the center of the open field box; *p* = 0.9238 and *p* = 0.7342, respectively. Outlier identified in the head-fixed group (black filled square at 30.166 s) but included in data analysis (removal would not change the statistical outcome). (**d**, **e**) Both groups had similar latency to the first floating event and spent similar time floating in the forced-swim test; *p* = 0.9838 and *p* = 0.6406, respectively. (**f**, **g**) Both groups traveled similar distances during the trial and spent similar time in the open arms of the elevated plus maze; *p* = 0.6711 and *p* = 0.9163, respectively. (**h**) Both groups built nests of similar quality using all the available nesting material; scale 1 to 5 (worst to the best quality score, respectively); *p* = 0.6263. (**i**–**m**) Two-bottle free choice task used to test sucrose preference; liquid consumption adjusted for the body weight. (**i**) No change in the sucrose preference score in the head-fixed group relative to controls; scale 0 to 1, 0 = 100% water and 1 = 100% sucrose preference; *p* = 0.1123. (**j**) No difference between the groups in the total volume of consumed sucrose (*p* = 0.9745). (**k**, **l**) Lack of statistically significant difference between the total volume of water vs. sucrose consumed in the head-fixed group (*p* = 0.2340) and a significant difference in the control group (***p = 0.0007). (**m**) Statistically significant difference between the groups in the total volume of consumed water (*p = 0.0486). Paired samples Student’s *t* test was used for all datasets except for (**e**), the total floating time where the Wilcoxon Single Rank test was used instead; n = 8 in each group.

Next, we examined if 25 days of 2-h head-fixed sessions produced any long-term behavioral effects. This information may be especially useful in the context of more complex behavioral paradigms that often require extensive training for several days or longitudinal studies on mechanisms of learning and plasticity^24,25^. For 4 days after the 25-day head-fixed protocol, we performed a battery of well-established behavioral tests known to be affected by chronic mild stress^3,26–28^. The test order was specifically designed to limit the confounding effect of potential stress evoked directly by the tests themselves (for details see "Methods", and the timeline presented in Fig. 3a). We started on day 26 in the morning with the open field test that is used to analyze general locomotor behavior as well as the anxiety/stress related to open space. We did not see any difference between the head-fixed and the control group in the total distance traveled (*p* = 0.9238; Fig. 3b). Also, the time spent in the center of the field was similar between the groups (*p* = 0.7342; Fig. 3c), as well as the number of returns to the central part of the field (*p* = 0.7662; Supplementary fig. S2a) and the latency to the first visit in the center (*p* = 0.6085; Supplementary fig. S2b). Six hours later, in the afternoon, we performed a forced-swim test that is used to assess instinctive survival behavior in extreme circumstances that may be affected by stress. We did not find any statistically significant change in the measured parameters: latency to the first floating event (*p* = 0.9838; Fig. 3d), total floating time (*p* = 0.6406; Fig. 3e), the number of floating events (*p* = 0.3891; Supplementary fig. S2c) and the number of feces left in the water (*p* = 0.3159; Supplementary fig. S2d).

In the morning of the second day of behavioral experiments, we performed an elevated plus maze test. This task relies upon rodents’ proclivity for dark, enclosed spaces by measuring their preference to spend time in the open or closed arms of the maze. Also in this task the head-fixed and control groups did not differ on any metrics (Fig. 3f, g and Supplementary fig. S2e–h), including similar overall distance traveled (*p* = 0.6711; Fig. 3f) and time spent in open arms of the maze (*p* = 0.9163; Fig. 3g). Before the onset of the dark cycle on this day we moved all the mice to individual cages with 3 g of compacted nesting material in each cage to measure the quality of their nests built the following morning using the scale standardized by Deacon^29^. This task is thought to provide an index of apathy/anhedonia which may be reflected by a lower quality of nest construction. We did not observe any group difference in nest quality (*p* = 0.6263; Fig. 3h). Both groups shredded the majority of the provided nesting material similarly and assembled it into a good quality nest, with the average score around 4.5 out of 5.0 points (4.500 ± 0.1336 in the head-fixed vs. 4.625 ± 0.1250 in the control group).

Another known measure of apathic/anhedonic behavior is sucrose preference, tested in the two-bottle free choice task. Thus, on days 3 and 4 of behavioral testing, the mice performed a two-bottle free choice task in which one bottle was filled with 1% sucrose and another with regular drinking water. In regular conditions mice, as in humans, show a preference for the sucrose solution^30^ that can be affected by stress. While there was no inter-group difference in sucrose preference (*p* = 0.1123), considerable variability was observed in the head-fixed group (the coefficient of variation in the head-fixed group was 46.86% vs. 15.24% in the control group; Fig. 3i). Also, sucrose intake measurements were more variable in the head-fixed animals as compared to the controls (61.62% vs. 19.69%, respectively; Fig. 3j). In addition, there was no statistically significant difference between the water and the sucrose intake in the head-fixed group (*p* = 0.2340; Fig. 3k) as opposed to the control group (*p* = 0.0007; Fig. 3l). Moreover, the head-fixed animals consumed significantly more water than the controls (*p* = 0.0486; Fig. 3m) suggesting a change in fluid intake that might relate to excess thirst in the head-fixed mice even a few days after the last head-fixed session.

### Locomotion pattern does not predict changes in the head-fixed stress, but distance traveled is correlated with corticosterone level

**Figure 4:**
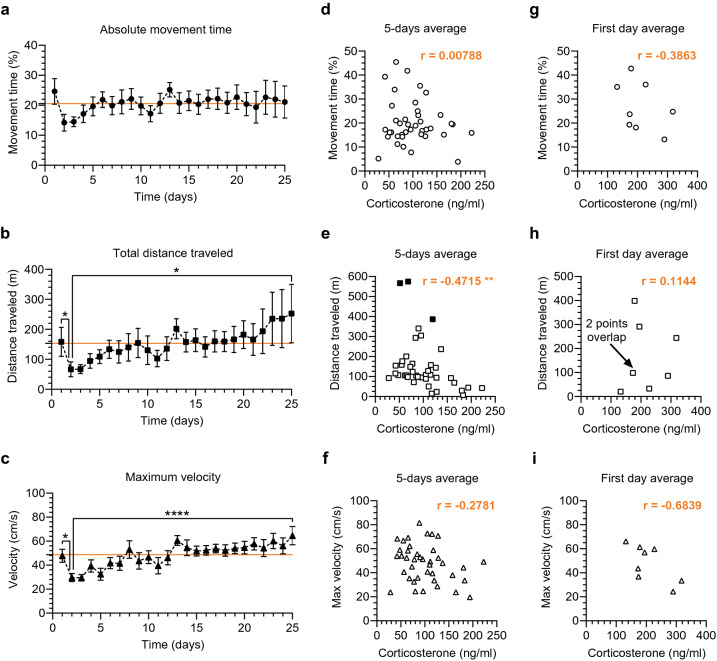
Locomotion dynamics and correspondence to the overall corticosterone level in the head-fixed animals. (**a**–**c**) Locomotion parameters in the 25-day protocol; daily averages calculated with a resolution of seconds; the solid orange lines correspond to the grand averages; data analyzed with the Friedman test followed by mean ranks comparison and Dunn’s correction. (**a**) Absolute movement time stabilized at about 20% after some initial fluctuations but no statistically significant effect of time (F_r_ = 30.86, *p* = 0.1578). (**b**, **c**) Significant effect of time in both the total distance traveled (F_r_ = 49.81, *p* = 0.0015) and in the maximum velocity (F_r_ = 96.18, *p* < 0.0001). Longer distance traveled and higher maximum velocity on day 1 (D1), drop on D2 and steady increase between D2 and D25 in both as well: D1 vs. D2 (*p = 0.0382 and *p = 0.0289, respectively); D2 vs. D25 (*p = 0.0382 and ****p < 0.0001, respectively). (**d**–**f**) Correlation between the blood corticosterone concentration and the locomotion parameters. Corticosterone level measured every 5 days during the 25-day head-fixed habituation protocol. Locomotion parameters presented as averages for the head-fixed sessions in-between blood sampling (e.g. day 2–5, day 6–10, etc.). (**d**) No statistically significant correlation between the blood corticosterone and the absolute movement time (r = 0.00788, *p* = 0.9615). (**e**) Statistically significant correlation between blood corticosterone and the total distance traveled (r = – 0.4715, **p = 0.0021). Outliers identified (black filled squares at 386.025, 566.660 and 574.568 m), but included in data analysis (removal would not change the statistical outcome). (**f**) No statistically significant correlation between blood corticosterone and the maximum velocity (r = − 0.2781, *p* = 0.0823). (**g**–**i**) Correlation between the blood corticosterone and the locomotion parameters from the first day of the head-fixed habituation (omitted in the analysis presented in **d**–**f**). No statistically significant correlation between the corticosterone and the absolute movement time, the total distance traveled and the maximum velocity (r = − 0.3863, *p* = 0.3446; r = 0.1144, *p* = 0.7873; r = − 0.6839, *p* = 0.0614, respectively). Comparisons in (**d**, **e**) made with the Spearman’s correlation analysis and in (**f**–**i**) with the Pearson’s correlation analysis; n = 8.

In both humans and rodents physical exercise, such as voluntary wheel running, promotes stress resilience and can reduce response to stressor exposure^31,32^. Head-fixed mice in the MHC can easily move the floating container with their paws, so we collected locomotion data to see whether movement in this setting is related to levels of the blood corticosterone concentration. We performed video tracking of a yellow sticker attached to the MHC container which provided a reliable report of the mouse’s body movement (Fig. 1e). Examination of movement-related heatmaps and track maps gave us a general idea about the mouse’s locomotion (Fig. 1f). Next, we characterized movement further by quantifying movement time, average velocity and total distance traveled during every session throughout the 25 days of training. We used the Friedman test followed by mean ranks comparison and Dunn’s correction. Statistical analysis did not reveal any significant difference in the absolute movement time (F_r_ = 30.86, *p* = 0.1578), although there were visible fluctuations during the 25-day head-fixed protocol (Fig. 4a). Mice were more active on the first day relative to subsequent days (24.61 ± 4.27% of the session time spent moving on the first day). The average movement time then significantly dropped on day 2 to 14.2 ± 2.78%. Finally, the percentage of the time spent moving stabilized on the day 5 at about 20%, remaining at this level with some minor fluctuations until day 25 (the grand average over 25 days was 20.5 ± 0.52% of the session time spent moving). In contrast, the total distance traveled as well as the maximum velocity per day consistently increased across habituation days after day 1 (Fig. 4b, c). Statistical analysis showed significant effect of time in both cases (F_r_ = 49.81, *p* = 0.0015 for distance and F_r_ = 96.18, *p* < 0.0001 for velocity). There was a clear novelty effect on day 1 represented by significantly longer distance and significantly higher maximum velocity on day 1 than on day 2 (*p* = 0.0382 and *p* = 0.0289, respectively). In fact, both parameters dropped on day 2 and only afterwards was the locomotion increased, as seen in comparisons between day 2 and day 25 (*p* = 0.0382 for the distance and *p* < 0.0001 for the velocity).

Next, we checked whether changes in locomotion parameters were related to decreasing corticosterone level. We did not find any significant correlation between the absolute movement time or maximum velocity and the corticosterone level (*p* = 0.9615 and *p* = 0.0823, respectively; Fig. 4d, f). However, there was a significant negative correlation between the distance traveled and the corticosterone level (**p = 0.0021; Fig. 4e). The longer the distance the animals ran, the lower the level of corticosterone. Furthermore, we also tested whether any of the locomotion parameters measured on the first day could be used as a predictor of the day 1 corticosterone level. We did not see any statistically significant correlation with the absolute movement time, the maximum velocity or the distance traveled (*p* = 0.3446, *p* = 0.7873 and *p* = 0.0614, respectively; Fig. 4g–i). These results suggest that the overall locomotion pattern cannot be used as a reliable biomarker of the stress level in the head-fixed mice.

### Dynamics of the voluntary locomotion behavior in the head-fixed mouse

**Figure 5:**
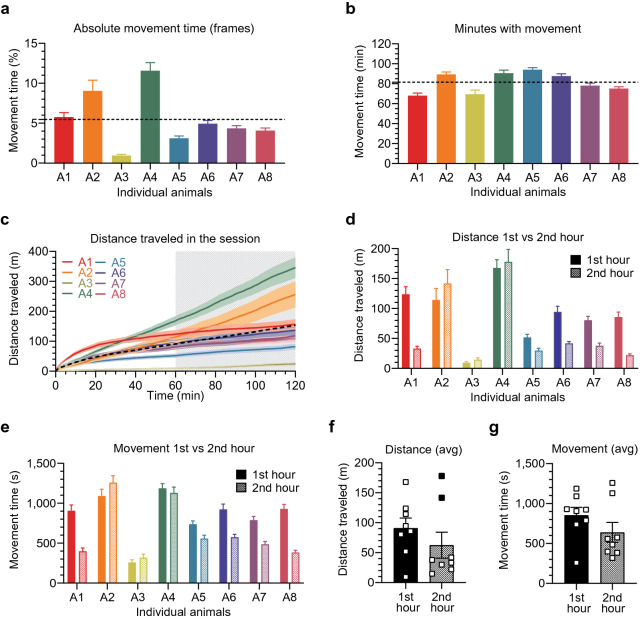
Locomotion dynamics during the 120-min head-fixed session. (**a**–**g**) Movement and distance analysis, averages from all the 25 head-fixed sessions. (**a**, **b**) Individual differences between the head-fixed animals in the absolute movement time (proportion of frames with movement recorded at 30 Hz resolution) and in the minutes with movement per head-fixed session (when counting every minute with any movement), respectively; dashed line represents the grand average from all the sessions and all the animals. (**c**) Average distance traveled during every minute of the 120-min head-fixed session, all days combined; dashed line corresponds to the grand average from all the sessions and all the animals combined. (**d**–**g**) Total distance traveled and the movement time during the first and second hours of the head-fixed session calculated with a resolution of seconds. (**d**, **e**) Individual animal data for the total distance traveled and for the movement time. (**f**, **g**) No statistically significant change in the total distance traveled and in the average movement time between the first and the second hour of the head-fixed session (paired *t*-test, *p* = 0.0882 and *p* = 0.0514, respectively). Outliers identified at 2nd hour (black filled squares at 141.643 and 177.890 m) but included in data analysis (removal would change the statistical outcome to significant drop in distance traveled during the 2nd hour of the session with *p* = 0.0222); n = 8.

In the head-fixed experiments, mice and rats are often trained to perform complex sensory-motor tasks while their brain activity is being monitored with sensitive recording techniques, such as patch-clamp electrophysiology^8,33^. Spontaneous voluntary movement, i.e. running in the MHC, may affect training efficiency as well as hinder neural recordings or interpretation of the results. Thus, determination of the optimal experimental design for understanding locomotion dynamics will be useful for experimenters, especially if they are interested in voluntary movement in this head-fixed setting. We found that mice were showing relatively short bursts of movement spread out over much of the duration of the session which was clear from the movement time analysis. The absolute movement time calculated on a per frame scale (the frame rate = 30 Hz) showed that, on average, the mice were moving 5.5 ± 0.32% of the 120-min session (Fig. 5a). At the same time, if every minute when some movement occurred is summed up, it was about 60–90 min in every session (Fig. 5b). Hence, the total distance traveled had been increasing constantly from the first to the last minute of the session (Fig. 5c). To learn whether there was any general trend as to when the movement occurred, we analyzed the first and the second hour of every session. We averaged all days for each animal separately, taking into account individual differences. It appeared that most of the animals were more active and traveled longer distances during the first hour (Fig. 5d, e). However, this difference did not reach statistical significance in the analysis of the cumulative average from combining all animals and all days together (*p* = 0.0882 for the distance and *p* = 0.0514 for the movement analysis; Fig. 5f, g), potentially due to high individual variability between the animals (the coefficient of variation, 1st and 2nd hour: 52.31% and 98.72% for the distance; 32.94% and 55.72% for the movement).

### Individual differences and motor skill refinement in the floating container

Moving the air-lifted floating container in a controlled manner requires practice that leads to improvement in speed, accuracy, and consistency of movement with training. Hence, it can be classified as a learned motor skill^32^. We observed refinement of this skill at a different pace for different animals, so we also quantified movement time of individual animals throughout 25 days of habituation. We found 3 types of animals: animals that increased, decreased, or showed no change in overall movement metrics across sessions (Supplementary fig. S3a–c, respectively). Nevertheless, all of them seemed to develop better control over the floating container with time. Thus, we went on to characterize the movement quality by focusing on changes in velocity in every session.

**Figure 6:**
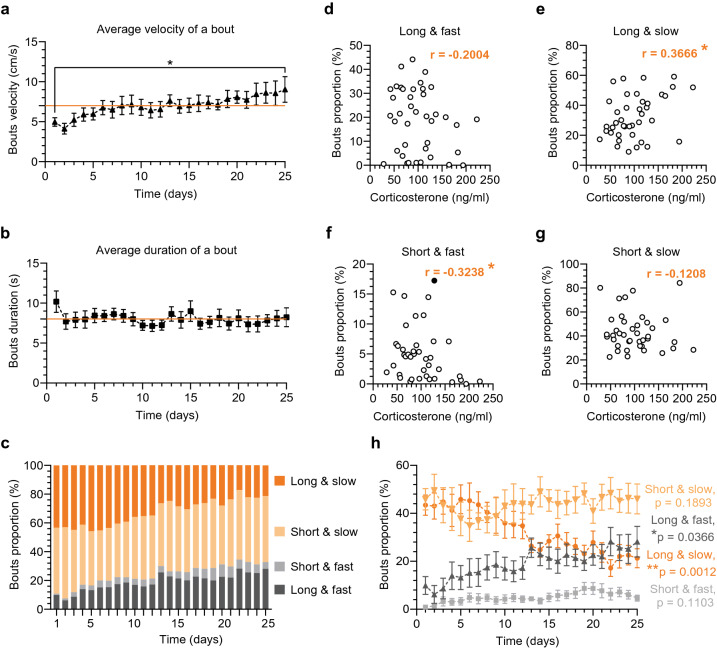
Motor control over the container movement and correlation with the blood corticosterone. (a–h) Detailed velocity analysis based on the bouts of activity (for details see “Methods”) during the 25-day head-fixed habituation protocol; solid orange lines correspond to the grand averages (all days and all animals together). (**a**, **b**) Data analyzed with one-way RM ANOVA followed by Bonferroni’s post hoc tests. (**a**) Statistically significant increase in the average velocity of individual bouts during the habituation protocol: F(2.669, 18.69) = 3.825, *p* = 0.0307 for the ANOVA, and *p = 0.0425 for the post hoc day 1 (D1) vs. D25 comparison. (**b**) Stable average duration of a bout throughout the habituation protocol with minor statistically non-significant fluctuations: F(3.301, 23.10) = 1.538, *p* = 0.2293 for the ANOVA. (**c**) Bouts of activity dynamics, changes in the proportion between the 4 different categories of bouts with passing time (for details see “Methods”). (**d**–**g**) Correlation analysis between the blood corticosterone concentration and changes in bout proportions: Spearman’s in (**d**, **f**, g**)**, and Pearson’s in (**e**). Corticosterone level measured every 5 days during the 25-day head-fixed habituation protocol. Bouts presented as averages for the head-fixed sessions in-between blood sampling (e.g. D2–D5, D6–D10, etc.). Higher corticosterone level corresponded to more of the long & slow movements (r = 0.3666, *p = 0.0200) and to less of the short & fast movements (r = − 0.3238, *p = 0.0415). However, no correlation was observed in comparisons with other types of bouts: long & fast (r = − 0.2004, *p* = 0.2151); short & slow (r = − 0.1208, *p* = 0.4577). (**h**) Statistically significant changes in the proportion of the long bouts with training time and no changes in the short bouts, one-way RM ANOVA: long & fast, F(2.990, 20.93) = 3.408, *p = 0.0366; long & slow, F(4.138, 28.97) = 5.920, **p = 0.0012; short & fast, F(3.085, 21.59) = 2.248, *p* = 0.1103; short & slow, F(4.352, 30.46) = 1.627, *p* = 0.1893; n = 8 for all datasets.

We observed that the majority of mice in the first session had difficulty controlling the floating container. With training time, these initial uncertain and random movements were replaced by organized exploratory behavior and smooth-running events, a sign of a more controlled movement technique. To quantify this transition, from velocity analysis we extracted *bouts of activity* that were defined as temporary changes in velocity as opposed to the resting time when a mouse did not move the floating container (Supplementary fig. S4a–c). We focused on two aspects that may be helpful in describing movement control namely duration and velocity of individual bouts. Improvement in movement control, was measured as significant increases in the average velocity of a bout (one-way RM ANOVA: F(2.669, 18.69) = 3.825, *p* = 0.0307; Fig. 6a), while there were no significant changes in the average duration of a bout throughout the head-fixed protocol (one-way RM ANOVA: F(3.301, 23.10) = 1.538, *p* = 0.2293; Fig. 6b). To characterize movements in detail, we also classified the bouts into four categories by their duration, short or long, and by their velocity, slow or fast (for details see “Methods” and Supplementary fig. S4d).

Interestingly, there was a similar proportion of short and long bouts throughout the 25-day protocol (on average 48.5 ± 0.92% and 51.5 ± 0.92%, respectively; Fig. 6c). However, what changed the most was the increase in the number of fast bouts, both long and short (9.9 ± 3.69% to 28.2 ± 6.39% and 0.9 ± 0.34% to 4.6 ± 1.26%, respectively) at the expanse of long & slow bouts (43.4 ± 4.22% and 21.3 ± 3.98%). The increase in fast bout proportion corresponded to mice having smoother movements, longer exploration, and faster running. Finally, we examined whether any of the aforementioned bout types were related to the corticosterone decreases during the 25-day habituation protocol. Indeed, we found a positive correlation between the corticosterone level and decrease in the long & slow bouts (*p = 0.0200, Fig. 6e), and a negative correlation with the increase in the short & fast bouts (*p = 0.0415, Fig. 6f). In contrast, the long & fast and the short & slow bouts were not significantly correlated to the corticosterone level (*p* = 0.2151 and *p* = 0.4577, respectively; Fig. 6d, g). Because the bout dynamics may be affected by training time, we also examined the relationship between training day and bout proportion. One-way RM ANOVA revealed statistically significant changes in the proportion of the long bouts with training time (long & fast, F(2.990, 20.93) = 3.408, *p = 0.0366; long & slow, F(4.138, 28.97) = 5.920, **p = 0.0012) and no changes in the short bouts (short & fast, F(3.085, 21.59) = 2.248, *p* = 0.1103; short & slow, F(4.352, 30.46) = 1.627, *p* = 0.1893) (Fig. 6h). Therefore, both the increased proportion of the short & fast bouts and the decreased proportion of the long & slow bouts may be considered a behavioral correlate of the lower corticosterone level. However, the change in the latter measure also depends on training time.

### Detailed analysis of container-spinning behavior and other control experiments

**Figure 7:**
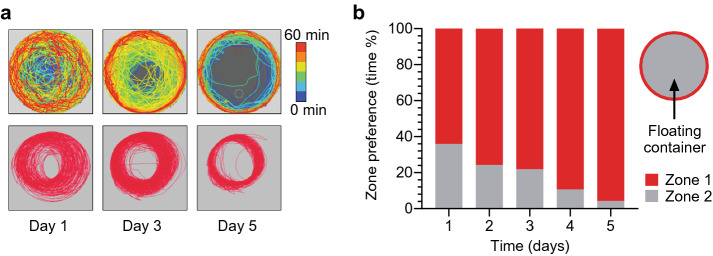
Spinning behavior analysis in the 5-day head-fixed protocol. (**a**) Example of locomotor activity measured with the MHC Tracking System (top panels) and with the EthoVision software (bottom panels). In the MHC Tracking System, the additional option of colorometric presentation was available (from blue colors to red colors reflecting the passing time of the session). Note the visible difference between the two methods. In the MHC Tracking System analysis, the lines represent movement of the container relative to the mouse. In the EthoVision software analysis – movement of the yellow sticker attached to the container. Analysis of spinning behavior in the context of a preferred placement of the container was possible only with the MHC Tracking System. Mice shifted the container from the middle to the side throughout the session and ran in proximity to the walls, once their control over the container was refined. (**b**) Zone preference data from all training days (D1–5) recorded from 4 animals presented as the average percentage of the time spent in the zone during the 60-min head-fixed session. Schema of the zone division of the floating container (red corresponds to the walls and grey to the middle part of the container).

In our study, the majority of mice began to *spin* the floating container as training progressed. During the early training sessions, the container was moved in all directions, but with time the mice gradually shifted to one side of the container and spent more time running in close proximity to the walls, moving the container in a single direction (a movement that resulted in the container spinning). We could not easily quantify this movement by tracking the yellow sticker attached to the container. Thus, we used the MHC Tracking System (Neurotar Ltd, Finland), a special system based on magnetic sensors designed for detailed locomotion analysis in the MHC set up (for details see “Methods”). In the 5-day protocol of our supplementary study (5 head-fixed sessions, 60-min each), we studied the spinning behavior. It was clear from the color-coded tracking maps that mice gradually developed controlled spinning behavior (Fig. 7a). They spent more time closer to the container walls as the time passed, within the session and throughout the training days. Furthermore, we extracted place preference data, dividing the container into two zones, the walls and the middle, to quantify the proximity to the wall (Fig. 7b). Indeed, mice spent more time in the wall zone than in the middle zone once they gained better control over the spinning movement.

**Figure 8:**
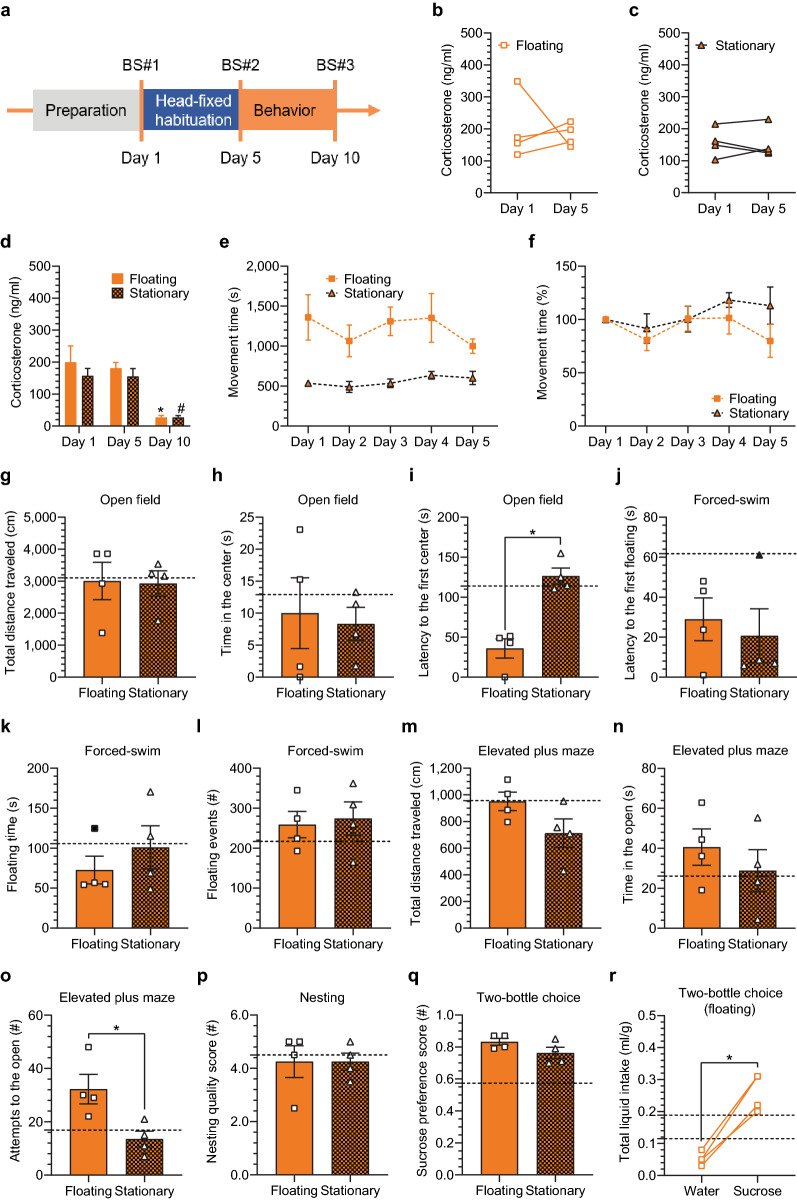
Corticosterone and locomotion dynamics in the 5-day head-fixed protocol followed by the stress-sensitive behavioral paradigms. (**a**) Experimental timeline: daily 60-min head-fixed sessions for 5 days followed by behavioral paradigms: the open field and the forced-swim tests (day 6, D6), the elevated plus maze (D7), nesting behavior (overnight D7/8), two-bottle choice (D8-9); two head-fixed groups: one with the air-lifted container (floating) and another with the container fixed to the base (stationary); blood sampling days (BS#). (**b**–**d**) Blood corticosterone concentration; n = 4 in each group. (**b**, **c**) Individual animal data. (**d**) Similar blood corticosterone level in both groups with a significant drop 5 days after the last head-fixed session at D10: two-way RM ANOVA followed by Bonferroni’s post hoc tests (within groups), interaction, F(2, 12) = 0.3431, *p* = 0.7163; time, F(1.374, 8.245) = 21.63, *p* = 0.0009; group, F(1, 6) = 1.041, *p* = 0.3470; a star (*) = significant difference in comparison to D1 only (*p = 0.0208) and a hash (#) to D1 and to D5 (#*p* < 0.04). (**e**, **f**) Movement time analysis; n = 3 in each group. (**e**) Significantly longer movement time in the floating group: two-way RM ANOVA, interaction, F(4, 16) = 1.719, *p* = 0.1950; time, F(4, 16) = 2.084, *p* = 0.1306; group, F(1, 4) = 10.80, *p = 0.0303. (f) Stable proportion of the movement time during the head-fixed sessions (normalized to D1). (**g**–**r**) Behavioral experiments analyzed with the Mann–Whitney U test; the dashed line corresponds to the head-fixed group from the 25-day protocol; n = 4 in each group. (**g**, **h**, **i**) Open field test: longer latency to the first center entry in the stationary group (Mann–Whitney U value (MWU) = 0, *p = 0.0286). (**j**, **k**, **l**) Forced-swim test: no differences in any presented parameters. (**m**, **n**, **o**) Elevated plus maze: less attempts to the open arm in the stationary group (MWU = 0, *p = 0.0286). (***p***) Similar nesting quality score between the groups. (**q**) High sucrose preference score in both groups (= sucrose preference). (**r**) Data from the floating group: higher sucrose intake (MWU = 0; *p = 0.0286).

In the 5-day protocol, we ran tests on two groups of animals: the *floating* group (the container was air-lifted and floating) and the *stationary* group (the container was fixed with tape to the base). The head-fixed sessions were shorter than in the 25-day protocol (60 min instead of 120 min) and we ran them for 5 days only (a timeline presented in Fig. 8a). Blood corticosterone concentration data for individual animals collected on day 1 and day 5 show that changes in the corticosterone level were variable in both groups (Fig. 8b, c). During the head-fixed habituation, the level increased in some cases and decreased in others. It seems that 60-min sessions were too short to significantly reduce the corticosterone level over 5 days. Interestingly, access to voluntary running in the floating group did not affect the corticosterone levels when compared to the stationary group, as concluded from the blood samples taken after the first and the last head-fixed session (Fig. 8d). Two-way RM ANOVA analysis revealed no group effect (F(1, 6) = 1.041, *p* = 0.3470) and no interaction between group and time (F(2, 12) = 0.3431, *p* = 0.7163). However, it showed a significant effect of time (F(1.374, 8.245) = 21.63, *p* = 0.0009) that was further tested with Bonferroni’s post hoc tests (within groups). Post hoc tests showed a significant drop in the corticosterone at day 10 (5 days after the last head-fixing) similar in both groups (day 1 vs. day 10, *p* = 0.0208 in the floating group; and *p* < 0.04 in the stationary group). We also analyzed movement during the head-fixed sessions. The floating group on average ran for 1217.0 ± 76.66 s out of 3600 s in each session, which was for significantly longer than the struggling body movements performed by the stationary group, on average for 559.3 ± 26.1 s (two-way RM ANOVA: interaction, F(4, 16) = 1.719, *p* = 0.1950; time, F(4, 16) = 2.084, *p* = 0.1306; group, F(1, 4) = 10.80, *p* = 0.0303)(Fig. 8e). However, in both groups the proportion of the movement time did not differ throughout training days (Fig. 8f).

After 5 days of head-fixing we conducted the same stress-related behavioral tests that were used in the 25-day protocol. These tests did not reveal any statistically significant differences between the floating and the stationary groups except for two minor parameters (Fig. 8g–r, Supplementary fig. S5a–h). In the open field test, the animals from the stationary group showed longer latency to visit the center of the box (Mann–Whitney U value, MWU = 0, *p* = 0.0286; Fig. 8i). In addition, they showed significantly lower numbers of attempts to enter the open arm in the elevated plus maze (MWU = 0, *p* = 0.0286; Fig. 8o). Both changes may reflect slight anxiety in the stationary group. Interestingly, in contrast to the 25-day protocol, the floating group expressed sucrose preference in the two-bottle free choice task. They showed a high sucrose preference score (0.8325 ± 0.02175 in the floating group vs. 0.5738 ± 0.09506 in the head-fixed group from the 25-day protocol; Fig. 8q) and higher sucrose intake in comparison to water (0.2600 ± 0.02915 ml/g for the sucrose vs. 0.05250 ± 0.01031 ml/g for the water in the floating group; MWU = 0, *p* = 0.0286), which convinced us that the fluid intake changes in the 25-day protocol may result from factors other than stress.

## Discussion

Measurement of blood corticosterone concentration is a commonly used method to assess physiological response to stress^34–36^. However, stress is a complex physiological mechanism incorporating changes in secretion of different hormones and transmitters, in addition to corticosterone (for review, see^4^). Therefore, any measurements of the blood corticosterone concentration related to particular stressors should be considered an indicator of potential stress, not an ultimate stress index. This is especially important to point out since the corticosterone level oscillates cyclically following the circadian rhythm in males and the estrus cycle in females^22,37^. In our studies, we counter-balanced groups for the circadian factor to be certain that what we recorded was related to head-fixation, not a natural circadian change in the corticosterone level. Furthermore, we used only males to avoid fluctuations related to the estrous cycle^22,37^ which would be especially difficult to overcome in the extended 25-day head-fixed protocol. Our head-fixed experiments revealed that the initial level of corticosterone in the blood sample after a single head-fixed session is almost 2–6 times lower^38^ in the MHC approach than in the full-body restraint, about 200 ng/ml vs. about 400–1200 ng/ml depending on the source of the ELISA assay^34^, the basal level of the corticosterone^39^ and the study design^22,28,40^. However, this value is still 9 times higher than the corticosterone level in the non-head-fixed control animals (about 20 ng/ml) which is lower or corresponds to the baseline corticosterone level observed in other studies^22,28,35,38–40^. Therefore, a habituation protocol where mice adapt to head-fixing constitutes an important experimental procedure, especially for experiments where stress is considered a confounding factor. After careful analysis of our corticosterone data we recommend a 10-day habituation protocol as a good solution if your research plan does not allow for the full 25-day or longer habituation protocol. At day 10 corticosterone level as well as mouse body weight stabilizes which is a sign not only of reduced stress level but also adaptation to the new environment.

We performed several behavioral tests, including open field, forced-swim, elevated plus maze, nest building, and sucrose preference tests, for 3 days following the 25 days of head-fixation. These tests are known to be affected by chronic mild stress^3,26–28^, but when performed on their own can be easily misinterpreted. Thus, we performed all these tests to compare and validate their results. At the same time, we wanted to preserve temporal proximity to the 25 days of head-fixation, which resulted in performing two tests per day for the first 2 days. The order of these tests was chosen to allow considerable time between testing and to avoid effects of very stressful tests (e.g. forced-swim test) on other tests. However, it is still possible that their proximity in time could be mildly stressful and influence results. Nevertheless, we did not find any statistically significant difference in the stress-sensitive behaviors after the 25-day protocol, apart from a change in the water intake revealed in the sucrose preference test. Neither did we see any difference after the shorter 5-day protocol. Both results were similar to a behavioral study performed on animals after full-body restraint^28^. It should also be noted that our control mice were exposed to mild stress throughout the pre-behavioral testing periods as well, in the form of routine exposure to the head-fixation facility room, the head-fixed cage mate, and repeated blood sampling. These environmental events might affect control mice behavioral metrics, making group differences more difficult to detect, yet we can still conclude that the daily head-fixation itself did not cause mice to perform these behavioral tests much differently.

We also studied voluntary locomotion during the head-fixed protocol, because increased stress level might affect learning of sensory-motor tasks as well as interfere with physiological recordings during their performance. We found that mice were generally active throughout 120-min sessions with slightly higher engagement in locomotion during the first hour. We also discovered strong individual differences between animals, suggesting 3 different types of mouse behavior: animal movement increases, decreases or no change. It may be interesting to follow up this distinction and assess whether these movement features correspond to other characteristics, such as learning capability or susceptibility to behaviors related to drug use disorders. Moreover, it had been shown that locomotion response to stress varies between sexes^41^. Thus, another direction for a new study based on the present data would be an in-depth analysis of sex differences in the stress response.

We examined the correlation between locomotion and stress indices as well. We found that corticosterone levels were generally lower with the larger numbers of rotations of the MHC container. Furthermore, we quantified movement refinement in the bouts of activity analysis in which uncontrolled movements were replaced by fully executed control over the container. This shift was reflected in the proportional change between different bout categories, namely: an increase in the long & fast and a decrease in the long & slow bout proportion. Notably, the change in the long & slow bouts was correlated with the level of corticosterone, but not the change in the long & fast bouts. In addition, the proportion of the slow & fast bouts was significantly correlated with the corticosterone level, but the short & slow bout proportion showed no such correlation. Hence, both the lower proportion of the long & slow bouts and the higher proportion of the slow & fast bouts may constitute reliable biomarkers for stress level assessment. Classification of movement by velocity (bouts of activity) may be a useful measurement for research dealing with motor skill learning and refinement. However, if we want to use these measures as reliable biomarkers, further work is necessary. In particular finding precise cut offs for bout classification and clear distinction between the “motor training effect” vs. the “corticosterone level dependence” is needed. This direction could be also extended to include different biomarkers of stress, especially given that there is abundant literature on endocrinological or immunological stress markers other than corticosterone, including levels of other hormones of the hypothalamic–pituitary–adrenal (HPA) axis (e.g. corticotropin-releasing factor, arginine vasopressin or adrenocorticotropic hormone), the size and the weight of the adrenal glands, the level of leukocytes, and the physiological response in the dexamethasone suppression test (DST)^20,27,28,42,43^.

It is important to note that there are many head-fixation methods^7,9,12,14^, and some, such as those in which animals cannot move^9,10^, are considered to be more stressful. We touched on that matter directly by comparing the floating and the stationary group in the final part of our studies, the 5-day protocol in the supplementary experiments. Nevertheless, it is only the beginning of more in-depth studies that need to be conducted to verify the role of locomotion in habituation to the head-fixed method. Our data from the 25-day head-fixed protocol should also be compared to data on corticosterone levels obtained with other body-restraining methods because of significant lack of literature on the stress evoked by the head-fixing itself. These comparisons suggest that locomotion while head-fixed may be a crucial aspect when aiming to reduce the stress level in the head-fixed setting. Also, it seems that the duration of the habituation protocol matters in this context. Our metrics relate primarily to the floating cage system. Meanwhile, other head-fixation techniques may show different stress-related physiological and behavioral dynamics. Therefore, further studies on this topic may be particularly useful for more-informed experimental choices.

In conclusion, we found that head-fixation is associated with at least one physiological sign of stress, namely increased blood corticosterone. Therefore, the stress-sensitivity of assays must be taken into account when designing experiments involving head-fixation. Nevertheless, the head-fixing procedure did not noticeably disrupt the refinement of movement or other behaviors either in the longer head-fixed protocol (25-day) or in the shorter one (5-day). Hence, the full 25-day habituation protocol does not have to be the best option for every type of experiment and shorter habituation protocols, run for 5 or 10 days, may be a reasonable choice. We believe that our attempt to fill an important gap in the current understanding of the head-fixed method may contribute to better experimental designs for in vivo experiments on head-fixed animals. We also hope that it will inspire other researchers to more closely examine this issue when analyzing and interpreting their results obtained in the head-fixed setting.

## Methods

### Animals

All animal protocols were approved by the US National Institute on Alcohol Abuse and Alcoholism (NIAAA). All experiments were performed in accordance with the National Institutes of Health (NIH) guidelines for animal research. All mice were purchased from the Jackson Laboratory (Bar Harbor, ME, US). Only adult, male mice were used in the matching age range, 13 weeks at the time of surgery, except for some animals used in the preparatory experiments (8 weeks) and the supplementary experiments (20, 21 and 24 weeks). This age-range was chosen because the younger, pre-adolescent animals present higher and more variable response to stressors^44,45^. C57BL6/J animals were used in the main project and supplementary experiments and the Emx1::Cre animals crossed with the Ai32 channel rhodopsin line were used in the preparatory experiments. We chose C57BL6/J mice for our experiments, because they remain the most commonly used animal model despite some limitations due to the use of this strain^16–18^. Our aim was to generate data that can be used as a point of reference for a wider audience. Choosing another strain generated by crossing the Emx1::Cre animals with the Ai32 channel rhodopsin line, on a C57Bl6/J background; that we used in the preparatory experiments was purely practical—these were the animals that were readily available in our laboratory at the time, and we plan to use them in future physiological experiments. All mice were housed on a 12-h light cycle (6:30–18:30), initially, 4 per cage. Immediately after the head-plate surgery, mice were separated and kept 2 per cage except for one head-fixed and one control animal (25-day protocol) that were single-housed and the animals in the preparatory study (5-day and 10-day preparatory protocol) that were always kept 2–4 per cage. Access to rodent chow and water was provided ad libitum*.* In total, 78 mice underwent head-plate-installation surgery, of which 3 died during surgery. Twelve animals were removed from the study due to head-plate loss during the experimental procedures and 11 of their littermates. 10 of these littermates (control animals) were used to study the influence of social isolation (single caging) on the corticosterone level. We found no difference in the blood corticosterone concentration in comparison to the animals that were paired-housed but significant effect of time: two-way RM ANOVA: interaction (F(5, 30) = 0.2453, *p* = 0.9406; group (F(1, 12) = 0.02966, *p* = 0.8661); time (F(5, 60) = 2.65, *p* = 0.0313); n = 7 in each group. Furthermore, we removed 1 animal due to tail injury (5-day preparatory protocol) and 6 additional animals that were planned for extra social isolation/blood sampling control experiments that were not included in this manuscript. All behavioral experiments were performed during the light phase of the day. Both the control and the head-fixed mice were tested in the same tasks during the same time of the day: in the morning, between 2 and 5 h from the beginning of the light cycle (Zeitgeber time, ZT), and in the late afternoon, between 10.5 and 11.5 h ZT). Animal cages were changed once a week by the animal facility caretaker on non-blood-sampling days and only after daily head-fixed sessions, to avoid an additional confounding stress factor. For the same reason, all the head-fixed sessions were run by the same experimenter. However, the blood sampling procedure was usually performed by another experimenter, so that the animals do not relate the smell of the person performing the head-fixed habituation to the stress generated by the head-fixed procedure.

### Head-plate surgery

Head-plate surgery was performed a week before the beginning of head-fixed experiments. Animals were placed in the stereotaxic frame under general isoflurane anesthesia (0.5–1.5% v/v isoflurane in oxygen). The skull was leveled by adjusting ear bar and nose bar position. The skull surface between the bregma and the lambda (cranial suture points) was exposed by removing a small piece of skin above this area with scissors and forceps. Next, the exposed skull surface was cleaned with 0.1 M phosphate buffer, then 70% EtOH and 3% Hydrogen peroxide and carefully dried with a Kim wipe. Head-plate model 8 or 9 that weighs about 1 g (Neurotar Ltd, Finland) was initially glued with superglue (World Precision Instruments, FL, USA) and secured to the skull with 2 miniature screws (Antrin Miniature Specialties Inc., USA) were fitted diagonally at a small angle next to the head-plate, so their heads stabilized the head-plate position. More glue was applied to a contact place between the head-plate and the skull. The connection was supported even further with dental cement (Coralite Dental Products Inc., USA). At the end of surgery animals were placed in new cages in pairs and a post-operative mix was applied daily for the following 2 days, Lactated Ringer’s Injection, USP (Hospira, Inc., IL, USA) at a dose 1 ml per 30.0 g of body weight.

### Head-fixed experiments

Mice were handled for 2 days in 15-min sessions per day. On the first day they could move around on the experimenter’s hands to get accustomed to the experimenter. On the second day they were exploring small pieces of cotton flannel of a similar white/light color to avoid potential color-bias (mice are able to see colors but with inability to distinguish some colors). Flannel pieces were assigned to individual mice to prevent another animal’s smell influencing the results. Mice were then wrapped and unwrapped in the flannel pieces several times during the handling session for proper habituation, as shown with the instruction videos available online (Neurotar Ltd, Finland). After 2 days of handling (one without the flannel and one with the flannel), the first day of the head-fixed protocol started. Each animal was weighed and then wrapped in the flannel. An animal from the head-fixed group was positioned in the head-fixed apparatus. The control animal was held in the flannel for a corresponding time then it was returned to its cage which was kept in the same room as their head-fixed littermate, so that they were always exposed to the same environment. The head-fixed apparatus was the Mobile HomeCage system (Neurotar Ltd, Finland) referred to as the *MHC*, that can also be built^46^. It is a research device where animals are head-fixed to an aluminum frame, but otherwise freely moving in an ultralight carbon fiber container floating above an air-dispensing base. The circular floating container used in this study with dimensions of 325 × 70 mm weighed about 50 g. Floating the container above the air-dispensing base was accomplished using air pressure from a connected pump (Matala Water Technology Co., Ltd., USA) adjusted to about 100–110 L/min at 2.9 psi.

Once the mouse was positioned under the aluminum frame, the head-plate was clamped in the head-post, the floating container was placed underneath, the flannel wrapping was removed, and the air-pump was turned on. The air-pump generated noise of about 70 dB and the MHC stage lighting was about 200 lx. The MHC stage was surrounded by non-transparent, white plastic walls that separated it from the rest of the room. There were several types of head-fixed protocols used in these studies including 5-day and 10-day preparatory experiments, the 25-day protocol in the main study and a 5-day protocol in the supplementary study. In all cases the head-fixed sessions were performed on consecutive days and lasted for 120 min except for the 5-day protocol in the supplementary study in which it lasted 60-min. In all the preparatory studies and 25-day protocol two groups were compared. The head-fixed and the non-head-fixed animals underwent similar procedures including the head-plate surgery. In the 5-day protocol in the supplementary study all the animals were head-fixed, but the comparison was between the *floating* group (the container was air-lifted and floating) and the *stationary* group (the container was fixed to the base). Furthermore, in the floating group the MHC Tracking System (Neurotar Ltd, Finland) was used together with magnetic tracking mats inserted in the container increasing its weight to about 65 g. The air-pump pressure had to be adjusted to about 120 l/min at 2.9 psi to make the container float in this condition. The container should be lifted by the air to about 0.1 mm above the base to reduce friction and allow for movement. If the air-pressure is too high, the mouse cannot control the movement of the container and the bottom part of the body constantly moves sideways. To standardize air-pressure between experiments a sheet of paper should just fit in-between the base and the container with no extra space. In the stationary group we used the same container with the inserted magnetic mat. The head-fixed apparatus, a head-fixed mouse and a flannel-wrapped mouse are shown in Fig. 1a–c.

### Corticosterone blood concentration measurement

After careful consideration of different blood sampling methods (for review, see^35,36^), tail vein bleeding from the awake animal was chosen. It was the least stressful procedure which allowed us to collect relatively large blood samples in a short time. It was easy to repeat several times in a consistent manner and helped to avoid the confounding effect of anesthesia. Blood sampling was performed immediately after the head-fixed session and each blood sample was about 40–50 ul. Mice were placed on the table under a small paper cup covering its body with the tail kept outside the cup stretched with a thumb and an index finger (Fig. 1d). The tail was snipped with a razor blade; a sample was collected with a heparin-coated glass capillary tube (Thermo Fisher Scientific, MA, USA) that was closed with the Surgipath Critocap (Leica Biosystems Richmond, Inc., IL, USA) at the end. The vein was secured afterward by applying gentle pressure with fingers above the cut while touching it with the Flexible caustic applicator 6″ coated with silver nitrate (75%) and potassium nitrate (25%) (Bray Group Ltd., England). The entire blood sampling procedure took no longer than 5 min (most often less than 2–3 min) minimizing confounding stress-response related to the blood sampling itself. It was reported that the corticosterone level increase in response to the stressor is noticeable no earlier than 5 min after its occurrence^35^. The sample was taken first from the control animal that had remained alone in the cage, then from the head-fixed littermate which was just coming back to the cage to avoid potential acute stress related to a bleeding littermate. The capillary with a blood sample was placed in the DM1424 Hematocrit Centrifuge (SCILOGEX, LLC, CT, USA) and spun at 15,000 g for 5 min at room temperature to separate the components of blood: red blood cells, platelets and plasma. About 15–20 ul of plasma that precipitated as a transparent uncolored layer was transferred to an Eppendorf tube and placed at − 20 C freezer for storage up to 4 weeks before further processing.

Blood corticosterone concentration was measured with enzyme-linked immunosorbent assay (ELISA, Enzo Life Sciences, Inc., USA) following the manufacturer’s instructions. The experimenter that ran the ELISA tests was blind to the samples to avoid any bias. The Enzo Life Sciences ELISA kit was preferred over other kits because it was showed the highest sensitivity in a previous study^34^. Corticosterone was chosen over cortisol as a stress marker due to its favorable dynamics corresponding more directly to the chronic stress response^22^. The assay was performed in duplicate and plates with final samples after running all the procedures were read on PHERAstar FS, a multi-mode microplate reader (BMG LABTECH Inc., NC, US). Open-access online analysis service (MyAssays Ltd., USA) was used for plotting the standard curve and data extrapolation. *Blood corticosterone concentration/corticosterone level* refers to the amount of corticosterone in plasma and is always expressed in nanograms per milliliter (ng/ml).

In the 10-day preparatory experiments, 25-day protocol and 5-day protocol in the supplementary study single blood samples were collected at the end of the session on the first day and every 5 days until the end of the experimental protocol. Furthermore, in the 25-day protocol additional control samples were collected 5 and 35 days after the last head-fixed session and in the 5-day protocol in the supplementary study, 5 days afterwards. Also, control experiments for the blood sampling procedure were performed by running 5 pairs of the head-fixed/control animals in the 25-day protocol and withdrawing them from the study at different times after a single blood sample performed on day 5, 10, 15, 20 and 25. Moreover, 8 single-housed animals underwent the same preparatory/surgical procedures and the full 25-day protocol without head-fixing and with the same 5-day blood sampling procedure for comparison with the pair-housed animals that were never head-fixed and were used as controls in the 25-day protocol. In addition, in the 5-day preparatory studies, 3 pairs of the head-fixed/control animals were blood sampled 3 times per day for 5 days.

### Behavioral tests

The effects of extended head-fixation were tested in classical stress-sensitive behavioral paradigms that are known to be affected in particular by chronic mild stress^3, 26–28^. The experimenter who ran these tests was blind to the experimental condition of the animal (the head-fixed or the control group) to avoid any bias. Behavioral testing started after the last day of head-fixation in the 25-day protocol and in the 5-day protocol in the supplementary experiments. Tests were performed on individual animals in the following order: morning of day 1—open field test (OFT); afternoon of day 1, about 6 h later—forced-swim test (FST); morning of day 2—elevated plus maze (EPM); day 2/3 overnight—nesting behavior test (NBT) and habituation to two-bottle choice (TBC) used for the sucrose preference test (SPT); day 3/4—SPT; day 5—control blood sampling. We designed this schedule to minimize stress evoked by the behavioral paradigms themselves and the potential carry-on effect between the tests. The most stressful FST was performed in the afternoon, between OFT and EPM, so that the animals had time to recover overnight. Additionally, setting up both the least stressful OFT and EPM in the morning before and after FST warranted cross-validation in the context of the fear of open space, the crucial parameter reflected in metrics of both paradigms. We used the cage-changing time during the NBT procedure as an opportunity to habituate animals to TBC to reduce the number of cage changes—another potential stress-inducing factor.

Mice were tested individually in all the behavioral tests. In OFT and EPM, all the surfaces were cleaned with 70% ethanol in-between running individual animals. In OFT, FST and EPM mice were transferred to the experimental room about 45–60 min before the beginning of the test. The same video camera Logitech HD Pro Webcam C920 (Logitech International SA, Switzerland) and the same video tracking analysis software EthoVision XT 13 (Noldus, The Netherlands) was used for all the recordings in OFT, FST and EPM. In OFT, the mouse was placed in the corner of a black plywood box without the top and the bottom (50 × 50 × 40 cm) standing on a light grey base. Every session lasted 10 min and was recorded with a video camera positioned above the center of the box. FST was performed in a transparent plastic cylinder filled with water (about 22–25 °C). Each animal was placed in the water for 6 min and movement/immobility was recorded with a video camera positioned approximately 2 m in front of the cylinder. After the test, the wet mouse was placed for about 15 min to dry on a paper towel in a holding cage warmed up with a heating pad to 37 °C. Then it was returned to its home cage. EPM consisted of two open and two closed arms (all arms: 30 × 5 cm) made of black plastic. The floor was made of white plastic for better contrast and it was elevated 50 cm above the ground. Closed arms had 15-cm-high black plastic walls on the sides and at the end. Each animal was placed in the middle of the EPM for 7 min and was recorded with a video camera placed above the center of the EPM. The first minute was skipped in the analysis to avoid random choices between the open and the closed arms in the early exploratory phase. NBT was performed following the guidance in the original paper by Deacon^29^. In short, mice were placed in new cages with 3 g of untouched nesting material and food pellets on the floor and they were left for the night. In the morning of the next day, the animals were transferred to new cages and the quality of the nests was ranked on a scale from 1 to 5 (the worst to the best quality, respectively). SPT was performed using a two-bottle free choice method in which each mouse was presented simultaneously with two bottles (25 ml). Initially, the animals were habituated overnight to the two-bottle choice (both bottles were filled with tap water and placed through the top of the cage lid). Then, the mice were transferred to new cages where one bottle contained tap water and the other 1% sucrose solution. After 24 h, the position of the bottles was switched to control for a side preference in drinking behavior. On the final day, the bottles were weighed, and the sucrose preference index was calculated based on the proportion between the volume of the liquids consumed.

### Locomotion analysis

Head-fixed mice in the MHC apparatus rested their paws on the air-lifted floating container. Thus, every movement of their paws corresponded to an immediate movement of the container that is easily recorded and tracked. Video recordings were collected with a high-definition camera Logitech HD Pro Webcam C920 (Logitech International SA, Switzerland) mounted above the head-fixed apparatus. Video-tracking software EthoVision XT 13 (Noldus, The Netherlands) was used to track the movement of a yellow sticker attached to the outer wall of the floating container. Movement of the floating container was used as an approximate measure for a mouse’s body movements/voluntary running in all the experimental protocols. In the stationary group of the 5-day protocol in the supplementary study, in which the container was fixed to an air-table, instead of the yellow sticker, mouse’s body movements were tracked directly (mostly movements of the paws and the tail) using the same tracking software.

In the floating group under the 5-day protocol in the supplementary experiments, the MHC Tracking System (Neurotar Ltd, Finland) was used in addition to the EthoVision software and the Logitech camera. The system consisted of a magnetic board installed inside the air-dispensing base and a rubbery mat with two built-in magnets inserted into the floating container. Air pressure was adjusted because the floating container with a rubber mat insert is heavier. Also, it was easier for a mouse to move the container with the insert because it had a much rougher surface providing an easier grip for a mouse. Changing position of the magnets during the head-fixed session was recorded and movement data were extracted with the accompanying software. This setup allowed for detailed analysis of spinning behavior (see “Results” for definition) as well. With the help of the accompanying software, the MHC container space was divided into two zones: a wall zone (area within 1 cm from the wall towards the middle of the floating container) and a middle zone (the rest of the inner space of the container). The time that the mouse spent in each zone during the head-fixed session was quantified. Moreover, it was possible to color-code their movement. The track maps corresponded to the time when the mouse was in a certain zone. The spectrum of colors from blue to red indicated the time into the head-fixed session, with blue used for the early times and red for the later times.

In the 25-day protocol motor skill refinement was quantified. Namely, transition from more random to organized and controlled movements was quantified with detailed velocity analysis. Temporary changes in velocity called *bouts of activity* were extracted and classified (for raw data, see Supplementary fig. 4a–c). There were 4 bout categories distinguished based on their duration and velocity: long & slow, long & fast, short & slow, short & fast. The cut off value for short-to-long duration was 5 s that corresponded to an average time in which mouse was able to move the container for one full lap/circulation. The cut off value for slow-to-fast velocity was 600 cm/min (about 10 cm/s), because it corresponded to the maximum velocity during the initial phase of learning before animals become efficient in control of the floating container (for raw data, see Supplementary fig. 4d).

### Statistical analysis

Data in the text and in the figures are presented as means ± SEM and the level of significance was *p* < 0.05 for statistical comparisons of all datasets. Outliers were identified in the same manner (ROUT method with Q = 1%) for all datasets and statistical tests were performed twice, once including and another excluding outliers. In most cases removal of outliers did not change the outcome of the statistical analysis, if otherwise, this is reported in the figure legend. Means and averages on the graphs are presented with outliers included in the final calculation. Parametric or non-parametric tests were chosen appropriately based on the normality of the data distribution. Data distribution was tested with the Shapiro–Wilk (W) test or by assessing distribution of residuals that were plotted on the graph. Single factor analysis for dependent samples was performed with the paired samples Student’s *t* test if data passed a normality test (*p* > 0.05) or with the non-parametric Wilcoxon Signed Rank test if they did not (*p* < 0.05). This testing was used in the behavioral experiments that followed head-fixation habituation (Fig. 3 and Supplementary fig. S2) and in the overall locomotion analysis (Fig. 5). The Mann–Whitney U test was used in the 5-day protocol in the supplementary experiments where the tested samples were smaller and normality assumption could not be tested appropriately (Fig. 8 and Supplementary fig. S5). When one factor was measured at different times for a single group, one-way RM ANOVA followed by Bonferroni’s post hoc tests was used when the data were normally distributed. The Greenhouse–Geisser correction for lack of sphericity in RM ANOVA was applied. When non-normal data distribution was indicated, the Friedman test followed by mean ranks comparison of preselected values with Dunn’s correction for multiple comparisons was used. One-way RM ANOVA and Friedman tests were used in locomotion (Fig. 4) and *bouts of activity* (Fig. 6) analyses. Two-way RM ANOVA followed by Bonferroni post hoc tests for multiple comparisons was used when one factor was measured at different times for two groups. Normal data distribution was indicated and the Greenhouse–Geisser correction was applied. This analysis was used for the corticosterone level analyses (Figs. 2, 8 and Supplementary fig. S1) as well as for body weight analysis (Fig. 2). Correlation analyses were performed to assess the relationship between two independent variables. If both datasets were normally distributed, Pearson’s correlation was used, otherwise Spearman’s correlation was used instead. This analysis was performed when correlating corticosterone levels with locomotion parameters (Fig. 4) or bouts of activity (Fig. 6). All data analysis was performed with GraphPad Prism 8.4.2 (GraphPad Software, CA, US).

## Supplementary information

Supplementary Legends.

Supplementary Figure 1.

Supplementary Figure 2.

Supplementary Figure 3.

Supplementary Figure 4.

Supplementary Figure 5.
