# Supplementary material for: Stress and behavioral correlates in the head-fixed method: stress measurements, habituation dynamics, locomotion, and motor-skill learning in mice
Source: Sci Rep. 2020 Jul 22;10:12245. doi: 10.1038/s41598-020-69132-6 (PMC7376196; doi:10.1038/s41598-020-69132-6)
Supplement: Supplementary file 1 — Supplementary Legends. [file 41598_2020_69132_MOESM1_ESM.docx]

**Supplementary information**

**Title:** **Stress and behavioral correlates in the head-fixed method: stress measurements, habituation dynamics, locomotion, and motor-skill learning in mice**

**Authors: *Konrad Juczewski^1^, Jonathan A. Koussa^1^, Andrew J. Kesner^1^, Jeong O. Lee^1^, and *David M. Lovinger^1^**

^1^Section on Synaptic Pharmacology & In Vivo Neural Function, Laboratory for Integrative Neuroscience, National Institute on Alcohol Abuse and Alcoholism, US National Institutes of Health, Rockville, Maryland, USA

*Corresponding Authors:
Konrad Juczewski konrad.juczewski@nih.gov
David M. Lovinger lovindav@mail.nih.gov
Laboratory for Integrative Neuroscience
National Institute on Alcohol Abuse and Alcoholism
National Institutes of Health
Rockville, MD 20852

**Supplementary figure 1. Corticosterone dynamics in the preparatory and the control experiments.** (a-j) Blood corticosterone concentration data. (a) Timeline of the 10-day preparatory experiments; blood sampling (BS) every 5 days (BS#1, BS#2, etc.) at the end of the 120-min head-fixed session; n = 3 in each group. (b) Increased level of blood corticosterone in the head-fixed group and significant drop at day 10 (D10) of the head-fixed habituation. Two-way RM ANOVA: two-way RM ANOVA, interaction, F(2, 8) = 0.8942, p = 0.4462; time F(1.179, 4.678) = 4.678, p = 0.1001; group F(1, 4) = 149.7, p = 0.0003. (c) Timeline of the 5-day preparatory experiments; BS performed 3 times at every head-fixed session: at the beginning (0 min), in the middle (60 min) and at the end (120 min). (d) Percentage change in the corticosterone level during the head-fixed session normalized to the baseline collected at the beginning (0 min); data presented as 5-day averages at every time; n = 2 in the head-fixed and n = 3 in the control group. (e-g) Data for individual animals at the time 0, 60 and 120 min. (h, i) Blood sampling control data from the 25-day protocol. Comparison between individual animals that were blood-sampled several times with the ones that were sampled only once at a specific time (several vs. single BS subgroups); n = 5 for each group (the head-fixed and the control group). (j) Effects of extended head-fixation on the circadian rhythm of corticosterone; data presented as 25-day averages every time. Time measured from the beginning of the light cycle (Zeitgeber time, ZT); n = 2 in each group every time.

**Supplementary figure 2. Complementary data for the behavioral tests run after the 25-day head-fixed protocol.** (a, b) Open-field test: no statistically significant differences between the groups in the number of returns to the central part of the open field box and the latency to the first visit in the center of the open field box; p = 0.7662 and p = 0.6085, respectively. (c, d) Forced-swim test: no statistically significant differences between the groups in the number of the floating events and the number of feces left in the water after the trial; p = 0.3891 and p = 0.3159, respectively. (e-h) Elevated plus maze task: the latency to the first visit to the open arms (p = 0.6968); no statistically significant differences between the groups in the number of returns to the open arms and to the closed ones (p = 0.5669 and p = 0.5000, respectively); the total time spent in the closed arms (p = 0.6808 and p = 0.8650). Paired samples Student’s *t* test was used for all datasets except for (g), the number of returns to the closed arms, for which the Wilcoxon Single Rank test was used instead; n = 8 in each group.

**Supplementary figure 3. Individual differences in the voluntary running in the 25-day habituation protocol.** (a, b, c) Changes in the movement time, daily averages from individual animals over 25 days. Data organized in 3 groups based on the overall trend in the individual mouse’s locomotor activity: increase, decrease and no change with time.

**Supplementary figure 4.** **Bouts of activity analysis – single animal data examples.** (a-d) All data were obtained with a 30-Hz frame-rate camera and modified for a final analysis to a resolution of seconds to avoid movement artifacts. (a-c) Changes in velocity dynamics during a 120-min head-fixed session throughout 25-day protocol (data from day 1, day 15 and day 25, respectively). Visible increase in the bout frequency as well as in the bout velocity. (d) Data used for distinction between the slow bouts and the fast bouts. Bouts were organized by their velocity in bins with 50 cm/min increments (e.g. 0-50 cm/min; 50-100 cm/min, etc.). Histogram representing all bouts from the first 5 days (in blue) and the last 5 days (in red) of the protocol and depicting their total number. Distribution of different velocities was much broader at the end of the 25-day protocol. The cut off value for the slow and the fast bouts was 600 cm/min (about 10 cm/s), because it corresponded to the maximum velocity during the initial phase of learning before the animals became efficient in the control of the floating container.

**Supplementary figure 5. Complementary data for the behavioral tests run after the 5-day head-fixed protocol.** Behavioral experiments analyzed with the Mann-Whitney U test; dashed line corresponds to the head-fixed group from the 25-day protocol; n = 4 in each group. (a) Open-field test: no difference in the number of attempts to the center. (b) Forced-swim test: no difference in the number of feces left in the water after the trial. (c, d, e) Elevated plus maze: no differences in any of the measured parameters: the latency to the first open arm, the number of attempts to the closed arm, and the time spent in the closed arms. (f, g, h) Two-bottle free choice task used to test sucrose preference; liquid consumption adjusted for the body weight. (f) Data from the stationary group: higher sucrose intake (MWU =0, *p = 0.0286). (g, h) No difference in the sucrose and in the water intake between the groups.
